# Use of a deep learning algorithm for non-mass enhancement on breast MRI: comparison with radiologists’ interpretations at various levels

**DOI:** 10.1007/s11604-023-01435-w

**Published:** 2023-04-18

**Authors:** Mariko Goto, Koji Sakai, Yasuchiyo Toyama, Yoshitomo Nakai, Kei Yamada

**Affiliations:** Department of Radiology, Graduate School of Medical Science, Kyoto Prefectural University of Medicine, 465 Kajiicho, Kawaramachi Hirokoji, Kamigyoku, Kyoto 602-8566 Japan

**Keywords:** Breast cancer, Dynamic contrast-enhanced MRI, Non-mass enhancement, Deep learning

## Abstract

**Purpose:**

To evaluate the diagnostic performance of deep learning using the Residual Networks 50 (ResNet50) neural network constructed from different segmentations for distinguishing malignant and benign non-mass enhancement (NME) on breast magnetic resonance imaging (MRI) and conduct a comparison with radiologists with various levels of experience.

**Materials and methods:**

A total of 84 consecutive patients with 86 lesions (51 malignant, 35 benign) presenting NME on breast MRI were analyzed. Three radiologists with different levels of experience evaluated all examinations, based on the Breast Imaging-Reporting and Data System (BI-RADS) lexicon and categorization. For the deep learning method, one expert radiologist performed lesion annotation manually using the early phase of dynamic contrast-enhanced (DCE) MRI. Two segmentation methods were applied: a precise segmentation was carefully set to include only the enhancing area, and a rough segmentation covered the whole enhancing region, including the intervenient non-enhancing area. ResNet50 was implemented using the DCE MRI input. The diagnostic performance of the radiologists’ readings and deep learning were then compared using receiver operating curve analysis.

**Results:**

The ResNet50 model from precise segmentation achieved diagnostic accuracy equivalent [area under the curve (AUC) = 0.91, 95% confidence interval (CI) 0.90, 0.93] to that of a highly experienced radiologist (AUC = 0.89, 95% CI 0.81, 0.96; *p* = 0.45). Even the model from rough segmentation showed diagnostic performance equivalent to a board-certified radiologist (AUC = 0.80, 95% CI 0.78, 0.82 vs. AUC = 0.79, 95% CI 0.70, 0.89, respectively). Both ResNet50 models from the precise and rough segmentation exceeded the diagnostic accuracy of a radiology resident (AUC = 0.64, 95% CI 0.52, 0.76).

**Conclusion:**

These findings suggest that the deep learning model from ResNet50 has the potential to ensure accuracy in the diagnosis of NME on breast MRI.

## Introduction

Dynamic contrast-enhanced (DCE) breast magnetic resonance imaging (MRI) is a widely used imaging modality for breast cancer screening in high-risk patients, the diagnosis of breast lesions, and breast cancer staging [1, 2]. The American College of Radiology (ACR) Breast Imaging-Reporting and Data System (BI-RADS) [3] is an internationally accepted quality assurance system designed to standardize the reporting and diagnosis of breast MRI. According to BI-RADS, contrast-enhanced breast lesions on MRI are first classified into three lesion types: focus, mass, and non-mass enhancement (NME).

NME on breast MRI includes a wide range of histologic appearances with some overlap in imaging findings between malignant and benign lesions [4]. Although radiologists make a diagnosis based on BI-RADS MRI, both the inter-reader concordance rate in interpretations of NME and the diagnostic performance to distinguish between benign and malignant lesions have been reported to be lower than those of masses, even by well-experienced breast radiologists [4–10]. Balzer et al. [7] reported that the primary cause for false-positive breast MRI findings resulting in unnecessary biopsies may be due to NME.

In recent years, multiple investigators have developed machine-learning methods for the quantitative characterization of breast lesions on clinical images [11], and deep learning using a convolutional neural network (CNN) has been shown to be a feasible method for diagnosis without the need to use predefined feature extraction algorithms. Residual Networks (ResNet) is a commonly selected CNN for the analysis of magnetic resonance (MR) images that has been shown to be capable of diagnosing breast mass lesions with high accuracy [12, 13]. Although the diagnosis of NME using deep learning is more challenging than that of masses, Zhou et al. [14] reported that ResNet50 showed better diagnostic performance than the radiomics method for differentiating malignant and benign NME on breast MRI.

Therefore, to clarify whether deep learning is a sufficient and clinically effective diagnostic aid, especially for residents or general radiologists, the present study aimed to evaluate the diagnostic performance of deep learning methods for distinguishing malignant from benign NME on breast MRI compared with the readings of radiologists with various levels of experience.

## Materials and methods

### Patients

The institutional review board approved this retrospective study and waived the need for written informed consent. Data were collected by reviewing the MRI reports in the electronic medical records at our university hospital between March 2010 and March 2013. The inclusion criteria were: patients initially interpreted as having NME over BI-RADS category 3 on the breast MRI report, histopathologically confirmed malignant NME via biopsy or surgery, or benign NME via biopsy or long-term follow-up (at least 3 years). Initial MRI reports were created by three board-certified radiologists with at least 2 years of experience in breast MRI. A total of 104 consecutive patients with 106 lesions presenting NME were identified. The exclusion criteria were patients receiving any prior treatment (*n* = 11), vacuum-assisted biopsy before the MRI examination (*n* = 6), or insufficient image quality (*n* = 3). Two patients had bilateral NME. Finally, 84 women with 86 lesions presenting NME were included in the retrospective analysis.

### MRI protocol

All breast MRI examinations were performed using a 1.5-T MRI system (Gyroscan Intera; Philips Medical Systems, Best, The Netherlands) with both breasts placed into the dedicated breast-holder and a four-channel phased-array coil. Before intravenous administration of contrast material, bilateral transverse fat-suppressed T2-weighted (FS-T2W) fast-spin echo images were obtained with the following parameters: repetition time (TR), 6000 ms; echo time (TE), 100 ms; field of view (FOV), 320 mm; matrix, 320 × 288; slice thickness, 3.5 mm; 40 sections; parallel imaging factor, 1.4; fat suppression on spectral adiabatic inversion recovery (SPAIR); and time of MRI data acquisition, 2.5 min. Next, axial diffusion-weighted echo-planar images (DWI) were obtained with the following parameters: *b* values of 0 and 1000 s/mm^2^; TR, 5600 ms; TE, 67 ms; FOV, 350 mm; matrix, 112 × 112 mm; slice thickness, 5 mm; 28 sections; parallel imaging factor, 2; and total imaging time, 3.0 min.

DCE MRI was performed using enhanced T1 high-resolution isotropic volume excitation (eTHRIVE) before and twice after bolus injection of gadolinium-based contrast agents (0.2 mL/kg at a rate of 2 mL/s), followed by a 20-mL saline flush using an automatic injector. The center of k space for both the early and delayed contrast phases was acquired at 90 and 300 s after contrast agent injection, respectively. The DCE MRI parameters were as follows: TR/TE, 4.6/2.2 ms; flip angle, 15°; FOV, 320 mm; matrix, 304 × 304 mm; interpolated slice thickness, 1.0 mm; 150 sections; parallel imaging factor, 1.8; fat suppression on SPAIR; and total imaging time of each phase, 56 s.

### BI-RADS reading session by radiologists

All MR images from the 84 patients were independently reviewed by one highly experienced radiologist (Reader 1; a board-certified radiologist with 15 years of experience in breast MRI), one general radiologist (Reader 2; a board-certified radiologist with 9 years of experience in general radiology and 6 years of training in general radiology, including breast MRI, with approximately 200 breast MRI experiences), and one radiology resident (Reader 3; 5 years of experience in radiology and 4 years of training in general radiology, including breast MRI, with approximately 50 breast MRI experiences). Each radiologist reviewed all breast MR images, including FS-T2W, DWI, and DCE MRI, as in the standard clinical breast MRI reading flow. All readers were informed only about the lesion location (affected side of and area where the lesion existed in the breast) and patient’s age and blinded to the mammography and ultrasound findings, initial interpretation of NME, and final diagnosis.

They assessed the degree of background parenchymal enhancement (BPE) (minimal, mild, moderate, or marked). The distribution (focal, linear, segmental, regional, multiple regions, or diffuse) and internal enhancement pattern (homogeneous, heterogeneous, clumped, or clustered ring) of each NME were also evaluated using BI-RADS MRI lexicons. Thereafter, the readers provided a final BI-RADS category assessment. For the decision regarding the BI-RADS category, BI-RADS 4 was divided into three subcategories adopted for BI-RADS mammography and ultrasound, and then all lesions presenting NME were classified into the following six categories: 2 (0% probability of malignancy), 3 (> 0%, ≤ 2%), 4A (> 2%, ≤ 10%), 4B (> 10%, ≤ 50%), 4C (> 50%, < 95%), and 5 (≥ 95%). The readers assessed the likelihood of malignancy for each NME and determined a final category based on the probability of malignancy defined by BI-RADS [3]. The presence of previously reported findings suggestive of malignant NME (e.g., segmental or linear distribution, heterogeneous, clumped, or clustered ring internal enhancement, wash-out kinetics, low apparent diffusion coefficient on DWI) led to a higher category assignment for each reader [15–18].

### Deep learning analysis

#### NME segmentation

**Fig. 1:**
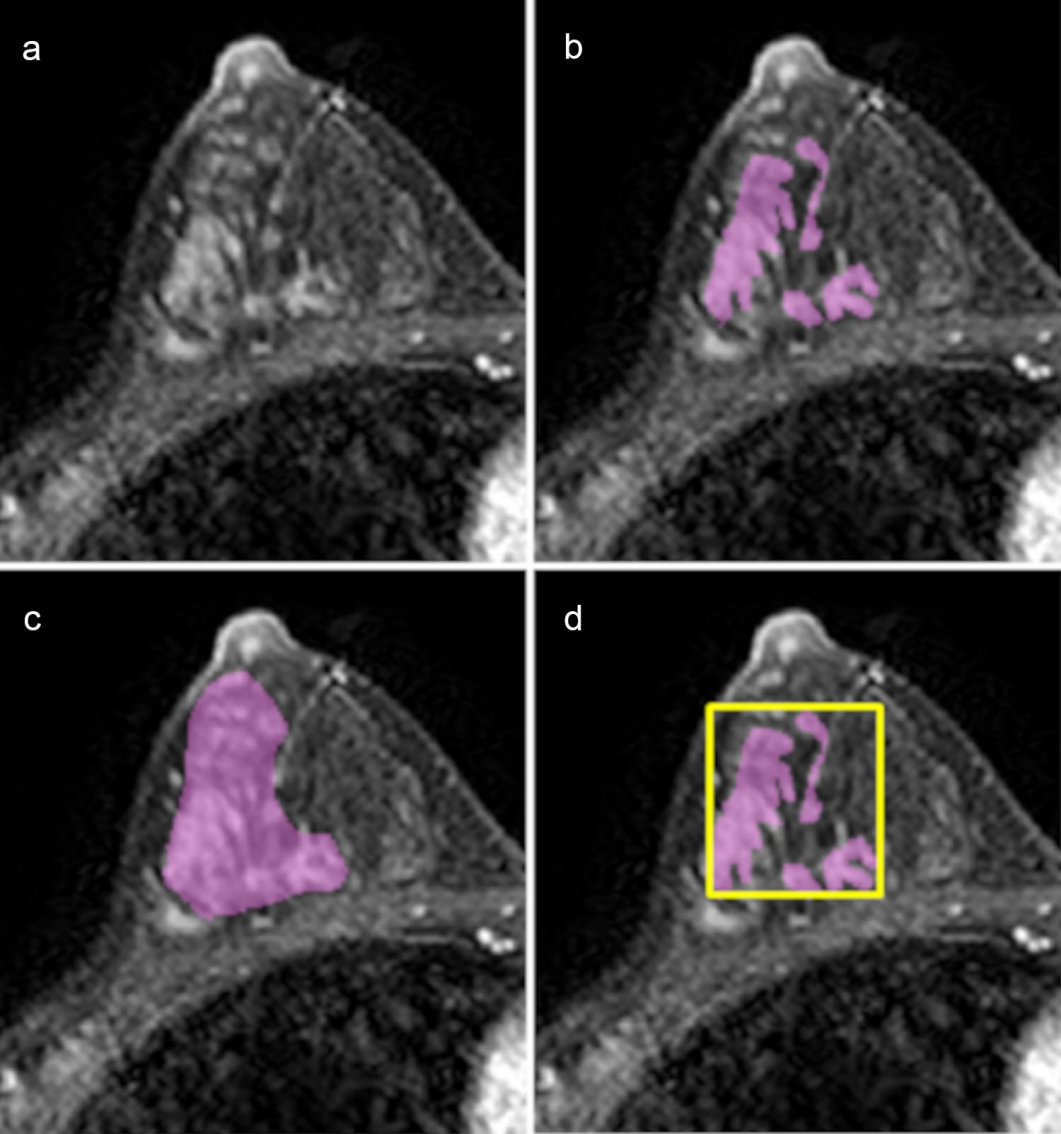
A 49-year-old patient with ductal carcinoma in situ. **a** Axial early-phase dynamic contrast-enhanced MRI demonstrates non-mass enhancement in the right breast. Reader 1 (highly experienced radiologist) assessed the lesion with segmental distribution and clustered ring internal enhancement, and finally decided on BI-RADS category 5. Two patterns of the ROI were set by one radiologist: **b** ROI-1, precise segmentation, which contains only the enhancing lesion, and **c** ROI-2, rough segmentation that covers the whole enhancing region, including the intervenient non-enhancing area. The input bounding boxes for ResNet50 were generated with the entire lesion **d** for all slices

**Fig. 2:**
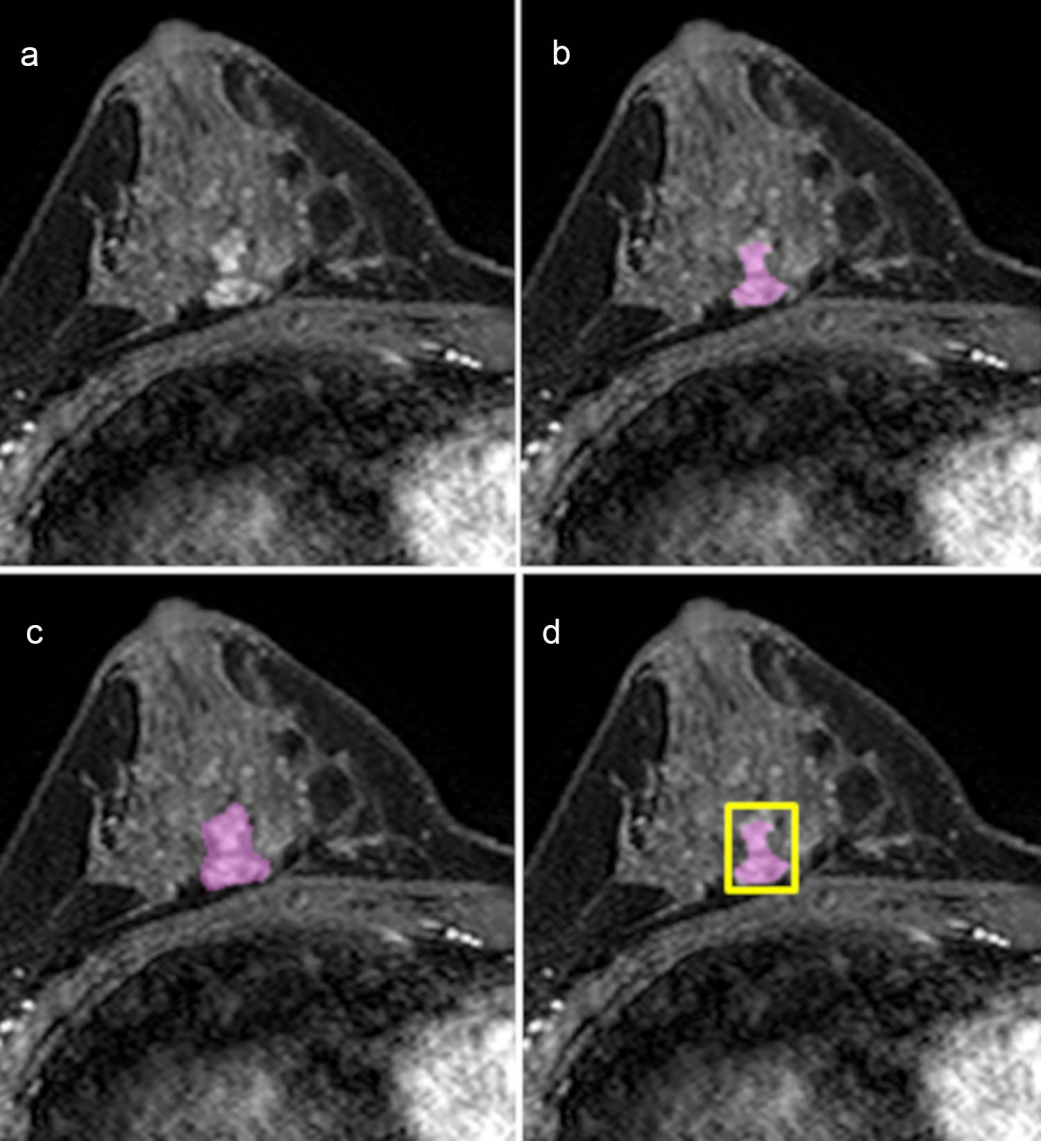
A 39-year-old patient with benign fibrocystic change. **a** Axial early-phase dynamic contrast-enhanced MRI demonstrates non-mass enhancement in the right breast. Reader 1 (highly experienced radiologist) assessed the lesion with focal distribution and clumped internal enhancement, and finally decided on BI-RADS category 4B. Two patterns of the ROI were set by one radiologist: **b** ROI-1, precise segmentation that contains only the enhancing lesion, and **c** ROI-2, rough segmentation that covers the whole enhancing region, including the intervenient non-enhancing area. The input bounding boxes for ResNet50 were generated with the entire lesion **d** for all slices

One breast expert radiologist (Reader 1) performed the NME segmentation, using the early phase (90 s) of DCE MRI on MRIcron (https://www.nitrc.org/projects/mricron). The lesion segmentation was done by Reader 1 prior to the BI-RADS reading session, and a sufficient period of at least 6 months was allowed between the reading session and NME segmentation. Three-dimensional regions of interest (ROIs) were manually drawn to cover the whole enhancing lesion in multiple sections. Two ROI patterns for each NME were set: ROI-1, a precise pattern that was carefully set to include only the enhancing area, and ROI-2, a rough pattern that covered the whole enhancing region, including the intervenient non-enhancing area (e.g., fat or normal fibroglandular tissues) (Figs. 1 and 2).

#### Deep learning method

**Fig. 3:**
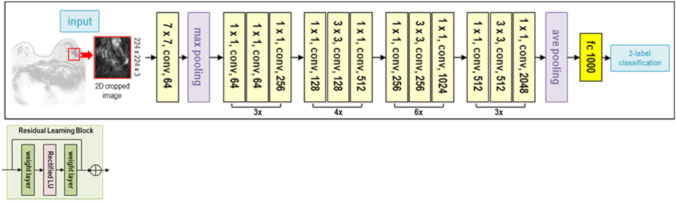
The ResNet50 architecture features a 3 × 3 convolutional (conv) layer, a max pooling layer, and 16 blocks of residual connections. Each block consists of three consecutive layers: a 1 × 1 conv layer, a 3 × 3 conv layer, and another 1 × 1 conv layer. The output from the start of the block is fed into the end of it through the residual connection. Finally, the output from the last residual block is connected to a fully-connected (fc) layer which uses a sigmoid activation to produce the final prediction

Deep learning was applied to differentiate the benign or malignant lesions automatically using pretrained ResNet50 architecture (Fig. 3) [19]. The software code was provided by MathWorks® (ResNet50) in the Deep Learning Toolbox. An Intel® Core™ i9-10900 K CPU (3.70 GHz) processor with 5 GB of NVIDIA GeForce RTX 3090 GPU graphics memory and 24 GB of physical memory running the education version of 64-bit Microsoft Windows 10 Home was used as the main experimental platform.

The inference model was created by DCE MRI, and the two-dimensional (2D) cropped images of pre-contrast, early, and delayed phases were used as inputs. For each case, a bounding box containing the entire lesion and covering the projected boundary was generated. This was done by projecting the segmented tumor ROIs from all slices together. The same box was used for all slices in one case. The input bounding boxes of the benign and malignant cases are shown in Figs. 1d and 2d. The bounding box was resized to 224 × 224 pixels as input into the networks. All tumor slices on all DCE MRI (6054 cropped images) were used as independent inputs, and the data set was not augmented. The loss function was cross-entropy. The training was implemented using the Adam optimizer fixed to 0.001 [13]. The diagnostic model was trained with a random selection of 70% of the data set. Tenfold cross-validation was performed to create the trained model. The classification performance was evaluated using 30% of the test data set. According to the probability of malignancy predicted in each slice, the results from all slices were combined to derive the receiver operating characteristic (ROC) curve.

### Statistical analysis

All statistical analyses were performed using JMP Statistics version 14.0.0 (SAS Japan, Tokyo, Japan, https://www.jmp.com) and R software (version 4.0.3; available as a free download from http://www.rproject.org).

The inter-reader reliability of the NME descriptions was examined by calculating the kappa coefficient, and those of the degree of BPE and the BI-RADS categorization were examined using the weighted kappa coefficient. A kappa statistic of < 0.4 was defined as poor agreement, that of 0.40–0.59 as moderate, that of 0.60–0.79 as good, and that of ≥ 0.80 as excellent.

The diagnostic performance for each reader and deep learning method was examined using ROC analysis and the area under the curve (AUC). The change in AUC was tested by DeLong’s test. All p values < 0.05 were considered statistically significant.

## Results

### Population characteristics

Among 84 women with 86 lesions presenting NME in this study, 51 had 51 malignant (mean age, 54 ± 12 years, range, 24–80 years) and 33 had 35 benign lesions (mean age, 49 ± 12, range, 21–74 years). The histopathological characteristics of the breast lesions are shown in Table 1.

**Table 1:** Histopathological characteristics of the 86 NME lesions

Malignant	51 (59.3%)
Ductal carcinoma in situ	29
Invasive ductal carcinoma	19
Mucinous carcinoma	3
Benign	35 (40.7%)
Fibrocystic change	17
Fibrosis	3
Fibroadenoma	1
Papilloma	3
Inflammation	1
Follow-up (over 3 years)	10

*NME* non-mass enhancement

### Radiologists’ assessments according to BI‑RADS MRI descriptors

Table 2 shows the results of the BI-RADS interpretations from each reader. Compared with the highly experienced radiologist (Reader 1), the less experienced radiologists (Readers 2 and 3) tended to interpret weak BPE. Regarding the NME descriptions, all of the following findings were correlated with the prevalence of cancer for all readers: segmental distribution (71.4%–84.6%) and clumped (66.7%–91.7%) or clustered ring internal enhancement (70.6%–93.3%).

**Table 2:** Interpretation of BPE and NME descriptors by each reader

	Reader 1		Reader 2		Reader 3	
	No. of lesions	Prevalence of cancer	No. of lesions	Prevalence of cancer	No. of lesions	Prevalence of cancer
BPE* (N = 84 patients)						
Minimal	47 (56)	–	68 (81)	–	70 (83)	–
Mild	18 (21)	–	11 (13)	–	10 (12)	–
Moderate	13 (15)	–	4 (5)	–	3 (4)	–
Marked	6 (7)	–	2 (2)	–	1 (1)	–
NME distribution (N = 86 lesions)						
Focal	37	40.5%	28	42.9%	20	35.0%
Linear	5	20.0%	11	63.6%	7	57.1%
Segmental	38	78.9%	26	84.6%	35	71.4%
Regional	0	–	9	44.4%	14	78.6%
Multiple regions	6	83.3%	3	100.0%	9	33.3%
Diffuse	0	–	9	33.3%	1	100.0%
NME internal enhancement (N = 86)						
Homogeneous	6	33.3%	18	33.3%	5	0.0%
Heterogeneous	55	49.1%	41	48.8%	43	58.1%
Clumped	19	89.5%	12	91.7%	21	66.7%
Clustered ring	6	83.3%	15	93.3%	17	70.6%

*Numbers in parentheses are percentages

*BPE* background parenchymal enhancement, *NME* non-mass enhancement

The prevalence of breast cancer by BI-RADS category for each reader is shown in Table 3. Reader 1 assigned four breast cancers to category 4A (cancer prevalence, 25%), which was above the BI-RADS-defined prevalence range of cancer for category 4A (> 2%, ≤ 10%). The cancer prevalence of Reader 1 in categories other than 4A was almost within the BI-RADS-defined rate.

**Table 3:** BI-RADS category and cancer prevalence for each reader

Category	Reader 1		Reader 2		Reader 3	
	No.of lesions	Prevalence of cancer	No. of lesions	Prevalence of cancer	No. of lesions	Prevalence of cancer
2	0	–	23	26.1%	1	0.0%
3	9	0.0%	15	40.0%	1	0.0%
4A	16	25.0%	7	57.1%	14	28.6%
4B	16	37.5%	12	75.0%	24	66.7%
4C	19	89.5%	11	81.8%	23	65.2%
5	26	92.3%	18	94.4%	23	69.6%

*BI-RADS* Breast Imaging-Reporting and Data System

In the BI-RADS categorizations of the two less experienced radiologists, Reader 2 tended to assign lower categories for each NME (cancer prevalence of category 2, 26.1%; category 3, 40.0%), while Reader 3 tended to assign higher categories.

Table 4 shows the inter-observer variability between the three radiologists. The BPE assessment between Readers 1 and 2 (*κ* = 0.41) and Readers 2 and 3 (*κ* = 0.42) showed moderate agreement. The agreement rate was particularly low for the NME distribution and internal enhancement characteristics of each reader, and the final BI-RADS assessment also showed poor agreement (κ < 0.40).

**Table 4:** Inter-reader agreement for BPE, NME descriptors, and category assessments

	Reader 1 vs. 2	Reader 1 vs. 3	Reader 2 vs. 3
BPE*	0.41	0.24	0.42
NME distribution	0.38	0.26	0.26
NME internal enhancement	0.25	0.24	0.15
NME BI-RADS category*	0.34	0.32	0.18

*BPE* background parenchymal enhancement, *NME* non-mass enhancement, *BI-RADS* Breast Imaging-Reporting and Data System

*Weighted kappa coefficient

### Diagnostic performance of deep learning methods

The diagnostic performance of deep learning constructed from two sets of ROIs (i.e., ROI-1 and -2) is shown in Table 5. When the models created from the training data set were applied to the test data set, the sensitivity, specificity, and accuracy were all better for ROI-1 than for ROI-2.

**Table 5:** Sensitivity, specificity, and accuracy using models constructed by ResNet50 deep learning

	Training data set (70%) 10-fold CV			Test data set (30%)			AUC
	Sensitivity	Specificity	Accuracy	Sensitivity	Specificity	Accuracy	
ROI-1	94%	83%	91%	95%	87%	95%	0.91
ROI-2	88%	72%	83%	89%	71%	89%	0.80

*ResNet50* Residual Networks 50, *ROI* region of interest, *CV* cross-validation, *AUC* area under the curve

### ROC analysis of the performance of deep learning vs. the radiologists

**Fig. 4:**
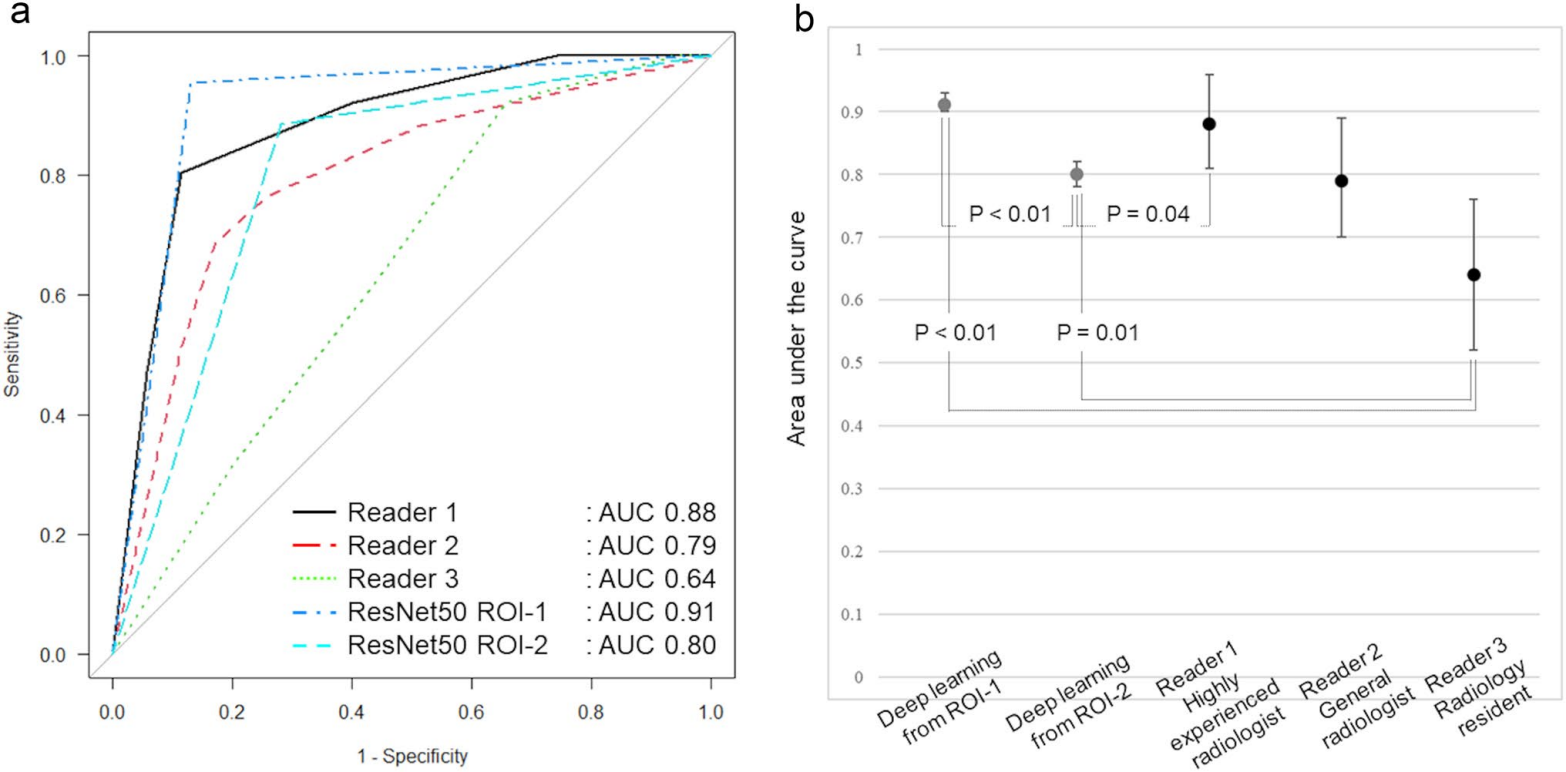
**a** ROC curves of BI-RADS in each reader and the deep learning in the test data set using ResNet50 ROI-1 and ROI-2. **b** Graph shows the AUC of the deep learning methods compared with various experienced radiologists. Error bars represent 95% confidence intervals. ResNet50 constructed from ROI-1 (precise segmentation) showed the highest diagnostic accuracy, following the highly experienced radiologist (Reader 1). ResNet50 constructed from ROI-2 (rough segmentation) showed diagnostic performance equivalent to the general radiologist (Reader 2), and both ResNet50 models had significantly higher diagnostic accuracy than the resident (Reader 3)

The evaluation using ROC analysis revealed that the performance of ResNet50 for ROI-1 [AUC, 0.91 (95% confidence interval (CI) 0.90, 0.93)] in the test data set was equivalent to that of the highly experienced radiologist [AUC, 0.89 (95% CI 0.81, 0.96), *p* = 0.45] (Fig. 4), whereas the performance of deep learning for ROI-2 [0.80, (95% CI 0.78, 0.82)] was significantly inferior to both (ROI-1, *p* < 0.01 and highly experienced radiologist, *p* = 0.04, respectively).

The AUC of deep learning for ROI-2 was similar to that of the general radiologist [0.79 (95% CI 0.70, 0.89), *p* = 0.88]. The AUC of the radiology resident [0.64 (95% CI 0.52, 0.76)] was significantly lower than that of both deep learning approaches (ROI-1, *p* < 0.01, and ROI-2, *p* = 0.01, respectively).

## Discussion

The present study confirmed the variability in the interpretation and diagnosis of NME on breast MRI owing to the radiologists’ experience. Deep learning methods have the potential to improve the accuracy of NME diagnosis and provide superior performance compared with readings from non-expert radiologists.

For masses, the BI-RADS MRI interpretation is known to be effective for predicting malignancy and shows good reproducibility for the final category assignment [6, 7, 10, 20]. However, for NME, the BI-RADS descriptors fail to diagnose correctly and several morphologic and kinetic features show an overlap of diagnostic information [6, 7, 20]. In the present study, the agreement rates between the three radiologists who had different levels of experience in general and breast radiology were poor for all BI-RADS MRI descriptors of NME (*κ* = 0.15–0.39). Previous reports have shown that there is significant variability among radiologists in choosing the optimal BI-RADS lesion description, especially when reporting non-mass lesions [8, 9]. The reported agreement rates were 0.25–0.27 in the distribution and 0.25–0.34 in the internal enhancement pattern [8, 9], which is consistent with the present study.

Tozaki et al. [8] also reported that the inter-reader agreement rate was poor for BI-RADS descriptors, but high for the BI-RADS category between the two raters who were experienced in interpreting breast MRI. However, in the present study, the agreement rates for the BI-RADS category and descriptors between the three radiologists with various levels of experience were also poor (*κ* = 0.18–0.34). As demonstrated in a previous study, the diagnostic performance of BI-RADS MRI reading was affected by reader experience [9, 10]; less experienced readers showed the poorest diagnostic outcomes in the interpretation of NME assessments [10], which is consistent with our results. In addition, subcategorization of BI-RADS category 4 lesions has not yet been adopted in MRI because of a lack of data on the accuracy of subdivision. Several studies have investigated category 4 subdivisions in MRI, but further validation is still needed for their feasibility [15, 18, 21]. This uncertainty in BI-RADS 4 subcategorization may have been one of the reasons for the poor inter-reader agreement in the present study.

Deep learning techniques are being introduced into the field of diagnostic imaging to resolve such differences in interpretation according to the experience of the radiologist [11]. In this study, for the purpose of assessing differences in deep learning performance by ROI setting, we constructed models using two ROI setting patterns for the same breast lesion, and then compared their performance to distinguish benign from malignant NME. As a result, the model from manual rough ROI-2 (AUC = 0.80), which contained substantial normal fibroglandular tissue or fat, showed significantly worse performance compared with manual precise ROI-1 (AUC = 0.91, *p* < 0.01), which contained only enhanced lesions. The peritumoral microenvironment of breast cancer is known to play an important role in tumor growth and invasion [22, 23], and peritumor tissue has been reported to provide helpful information for the diagnosis and prediction of prognosis on breast MRI [24, 25]. Zhou et al. [13] reported that deep learning methods with ROIs that included small amounts of peritumor tissue showed higher diagnostic accuracy than did ROIs using tumor alone for the diagnosis of mass lesions on MRI. They also reported that too much normal tissue reduced diagnostic performance, which is consistent with our results.

For machine learning-based diagnosis, precise lesion segmentation is a major issue because manual annotation by radiologists is both labor-intensive and time-consuming. Therefore, the technique cannot be applied to large data sets. In this study, even the model from rough segmentation ROI-2 showed equivalent diagnostic performance to the general radiologist (AUC = 0.79) and outperformed the radiology resident (AUC = 0.64, *p* = 0.01). These results may have important clinical implications for low-resource regions, where there are few radiologists specializing in breast imaging, because even this deep learning model from rough segmentation can be useful as a diagnostic aid for general radiologists.

In recent years, automated segmentation of breast mass regions on MRI has been investigated by deep learning [26–28]. By contrast, automatic lesion annotation of NME is very challenging because in NME, the tumorous tissues and stroma are mixed, and it frequently has more indistinct borders from BPE compared with masses. Therefore, a reliable automatic NME segmentation method has not yet been established. Further efforts toward the development of machine-learning techniques may improve the accuracy of NME identification and segmentation in the near future.

Wang et al. [29] reported that the diagnostic performance of deep learning in NME using maximum intensity projection (MIP) of early post-contrast subtracted breast MR images was lower than the clinical decisions made by senior radiologists based on the full diagnostic MRI protocol. In the present study, we constructed a deep learning model for breast NME using three-phase DCE MRI data as input, and found that the performance from precise ROI-1 was equivalent to that of highly experienced radiologists using a full diagnostic MRI protocol. These findings suggest that the performance of deep learning for NME could be improved by including the internal enhancement characteristics of DCE MRI, which are lost in 2D MIP images. Therefore, even the constructed diagnostic model using only DCE MRI data might achieve diagnostic performance equivalent to clinical decisions by highly experienced radiologists. Future studies are expected to improve the diagnostic accuracy of deep learning by inputting multiparametric MRI data.

This study had several limitations. First, the data set was comparatively small for the proper execution of deep learning. This mainly owes to the fact that there are far fewer numbers of NME compared with mass lesions. Thus, assembling a large NME data set with pathological confirmation, especially for benign cases, is difficult in real-life clinical settings. Second, this was a single-center study. The MR images were acquired using a single machine and a single protocol. Therefore, the study population does not include data from multiple vendors or images from different institutions. Accordingly, in the future, an external independent test data set is needed to evaluate the performance of the developed models. Lastly, manual lesion segmentation was performed by a single expert. Such radiologist-driven ROI approaches raise the concern of an inter-rater bias.

In conclusion, in the present study, we assembled a breast MRI NME data set, for which three radiologists with different levels of experience in breast imaging performed assessments of the morphological distribution and internal enhancement patterns based on BI-RADS MRI. The results showed poor agreement between raters. As expected, the less experienced radiologist showed the worst diagnostic performance. Deep learning methods were implemented to investigate their potential to diagnose NME, and high accuracy was achieved compared with the non-expert radiologists. Our deep learning model for diagnosing NME on breast MRI could therefore be a diagnostic aid for general radiologists.
